# The effects of cities on quail (*Coturnix coturnix*) migration: a disturbing story of population connectivity, health, and ecography

**DOI:** 10.1007/s10661-023-12277-4

**Published:** 2024-02-14

**Authors:** Jesús Nadal, David Sáez, Stefano Volponi, Lorenzo Serra, Fernando Spina, Antoni Margalida

**Affiliations:** 1 Department of Animal Science, Division of Wildlife, Faculty of Life Sciences and Engineering, University of Lleida, Avd. Alcalde Rovira Roure 191, 25198 Lleida, Spain; 2 Area Avifauna Migratrice, Istituto Superiore per la Protezione e la Ricerca Ambientale (ISPRA), Via Cà Fornacetta, 9, I-40064 Ozzano Emilia BO, Italy; 3 Institute for Game and Wildlife Research, IREC (CSIC-UCLM-JCCM), 13005 Ciudad Real, Spain; 4 Pyrenean Institute of Ecology (CSIC), Avda. Nuestra Señora de la Victoria, 12, 22700 Jaca, Spain

**Keywords:** Nature management, Anthropogenic homogenization, One Health for One Planet, Human pressure, Ecological connectivity, Migration vulnerability

## Abstract

The increasing impact of human activities on ecosystems is provoking a profound and dangerous effect, particularly in wildlife. Examining the historical migration patterns of quail (*Coturnix coturnix*) offers a compelling case study to demonstrate the repercussions of human actions on biodiversity. Urbanization trends, where people gravitate toward mega-urban areas, amplify this effect. The proliferation of artificial urban ecosystems extends its influence across every biome, as human reliance on infrastructure and food sources alters ecological dynamics extensively. We examine European quail migrations pre- and post-World War II and in the present day. Our study concentrates on the Italian peninsula, investigating the historical and contemporary recovery of ringed quail populations. To comprehend changes in quail migration, we utilize trajectory analysis, open statistical data, and linear generalized models. We found that while human population and economic growth have shown a linear increase, quail recovery rates exhibit a U-shaped trajectory, and cereal and legume production displays an inverse U-shaped pattern. Generalized linear models have unveiled the significant influence of several key factors—time periods, cereal and legume production, and human demographics—on quail recovery rates. These factors closely correlate with the levels of urbanization observed across these timeframes. These insights underscore the profound impact of expanding human populations and the rise of mega-urbanization on ecosystem dynamics and services. As our planet becomes more urbanized, the pressure on ecosystems intensifies, highlighting the urgent need for concerted efforts directed toward conserving and revitalizing ecosystem integrity. Simultaneously, manage the needs and demands of burgeoning mega-urban areas. Achieving this balance is pivotal to ensuring sustainable coexistence between urban improvement and the preservation of our natural environment.

**Supplementary Information:**

The online version contains supplementary material available at 10.1007/s10661-023-12277-4.

## Introduction

Although urbanized land covers less than 3% of the earth’s surface (He et al. 2019), the impacts of this artificial environment affect all ecosystems and their wildlife (Alberti and Wang 2022). The influence of mega-urbanizations, vast continuous urban areas, has seen a steady rise. These areas now produce 80% of the world’s gross domestic product (GDP) and accommodate 56% of the global population (World Bank, https://www.worldbank.org/en/home). Human encroachment continues to spread deeper into the remaining intact ecosystems and wilderness areas (Williams et al. 2020). Man-made infrastructures, toxic and greenhouse emissions, waste, and the agrosystems used to feed people and farm animals are affecting biodiversity and ecosystem services worldwide (Pickett et al. 2016). The locations, size, connectivity, and activities of mega-urbanized areas have spread to influence every ecosystem, to the point that One Health for One Planet initiatives require urgent implementation (Barroso et al. 2023; Fastré et al. 2021; Yin et al. 2022). For example, migratory birds must cross these altered biomes to complete their biological cycles and their survival depends on their relationships with ecosystems under pressure (Pancerasa et al. 2022). Indeed, ecosystems, agrosystems, and mega-urbanizations are now so entangled that the significant flow of materials and organisms between them creates widespread disturbances, such as changes in pollutants, diseases, or predation rates (Krauze and Wagner 2019).

Human history connects closely to both migration and environmental condition (Lainé and Morand 2020; Morand 2020). Biomes integrate both artificial drivers and constraints, followed by biodiversity loss (Secondi et al. 2020). Ecosystems and their services face substantial impacts from mega-urbanization, altering their natural functioning. The current state of ecosystems and agrosystems raises concerns about their performance (Alberti 2015; Lin et al. 2023). Many are operating below optimal levels due to these influences. Moreover, the permeability of cities for the passage of migratory birds remains an ongoing question (Alberti et al. 2017). Migration is the movement of a population from one biome to another in the spring with a return journey in the autumn, so as to maximize access to resources (Deboelpaep et al. 2022). This ecological strategy, so widely used by so many species and populations, makes migratory birds excellent indicators of ecosystem health over many biomes (Ogden et al. 2014). Studies of migrants also allow us to understand how the activities of mega-urbanizations are altering the planet (Alberti and Wang 2022).

The quail (*Coturnix coturnix*) stands as one of the  many migratory species that has not undergone a significant population decline in recent times (Nadal et al. 2020). It serves as a crucial ecological indicator, offering insights into the impacts of mega-urbanization on various biomes. This species boasts an ancient phylogenetic origin (Kimball et al. 2021; Wang et al. 2017), sharing migratory patterns, behavioral traits, and genetic characteristics with numerous contemporary Afro-Palearctic bird species (Haest et al. 2020).

With its migratory night flights, the quail adeptly navigates natural barriers such as deserts, seas, and mountains (Hedlund et al. 2022). Its protandrous nature sees males arriving at breeding grounds ahead of females. The population renewal occurs through successive waves of cohorts, enabling multiple reproductive attempts and maintaining high productivity (Nadal et al. 2018, 2019; Nadal and Ponz 2015). The historical use of the quail by humans for sustenance dates back so far that ancient records exist detailing its migratory behavior (Kennedy and Grivetti 1980), highlighting the longstanding relationship between human societies and this migratory bird.

Before the Second World War, a substantial portion of the population resided in rural areas. The post-war era witnessed significant economic growth fueled by the rise of urban industries. More recently, globalization has spurred a transformation of the land, resulting in profound changes to both rural and urban ecosystems. Each of these historical periods has indeed reshaped ecosystem dynamics, marking significant shifts in how urban environments interact with and impact other ecosystems. The exploration of these historical eras holds considerable significance in understanding the evolution of urban development, agricultural practices, ecosystem health, and the patterns of production and recycling.

The quail migratory network comprises the interconnected pathways and points utilized by birds during migration to traverse a geographic region. The western, central, and eastern Mediterranean regions serve as nodes in the European quail migratory network. The central node is located in Italy and is connected to nodes in North Africa, the Mediterranean, and Europe. We examined the history of quail migration along the central Mediterranean route and network using long-term data. We looked at the time periods between 1939 and 1942 before the Second World War (WWII), 1943–1951 after the war, and the present day 1998–2017 to determine how much mega-urbanization has affected the migration, population, ecosystem integrity, and health (i.e., the spread of disease between biomes) of quail. Human activities change the weather and natural cycles, affecting both ecological and agricultural production, and have an impact on migration routes. For example, artificial light at night (ALAN) has a strong influence and has increased by 9.6% annually in recent decades (Kyba and Newhouse 2023). The study of a migratory network can reveal migration routes and directions, population connectivity, barrier effects, and the transmission of diseases.

Urbanized areas contain a number of physical barriers such as buildings, towers, cranes, wires, and fences, and biophysical barriers such as ALAN, noise, air, and chemical (odors) and thermal pollution (Zuluaga et al. 2021). Quail can become disoriented when flying through urban areas, and collide with objects, or lose control in flight (Wiltschko et al. 2010). Quail can also become confused by early morning artificial light or be dazzled as they pass through an urban environment (Wilson et al. 2021). The failure of a migration route can cause population and ecosystem problems. Mega-urbanizations increase the likelihood of a night flight crash (McLaren et al. 2018). We hypothesized that over time, the urban barrier effect on quail migration has become increasingly severe in central Europe as mega-urbanizations increase and spread (Hahs 2016).

The quail population uses a variety of migration strategies (Brown et al. 2021), and includes individuals with both long and short migration phenotypes (Aoki et al. 2021; Handby et al. 2022). Social learning (Byholm et al. 2022), assortative mating (Berthold et al. 1992), innate and learnt behaviors (Burnside et al. 2020), large individual variation (Wong et al. 2022), and the tendency to rush across landscape barriers (Lathouwers et al. 2022) are characteristic attributes of this species. The conditions for suitable stopover and recuperation sites vary depending on the ecoregion (Herbert et al. 2022; Mackell et al. 2021; Schmaljohann et al. 2022; Wright et al. 2018). In summary, quail migration is influenced by both biological (internal) and ecological (external) factors.

## Methods

### The quail migratory network

**Fig. 1:**
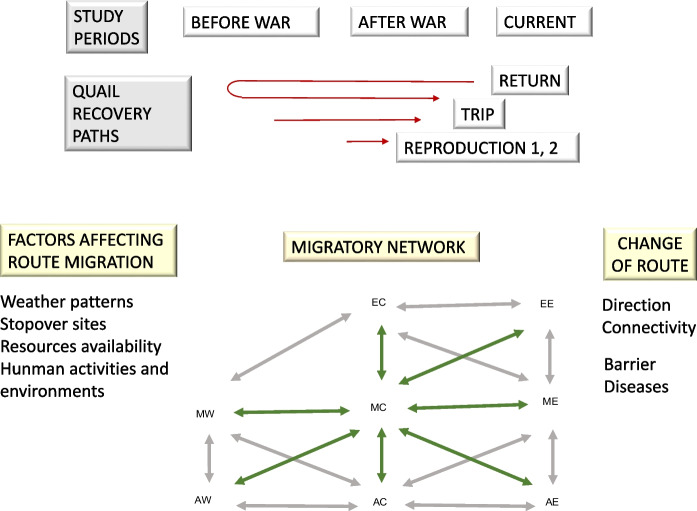
Diagram showing the migratory network of quail, the factors influencing it, and causes/consequences of route change. Migratory network nodes: MW, Mediterranean West; AW, Africa West; EC, Europe Central; MC, Mediterranean Central; AC, Africa Central; EE, Europe East; ME, Mediterranean East; AE, Africa East; arrows as connections between nodes. Data collected from three study periods and three recovery paths

To study the quail migratory network in the years before and after WWII, and in the present, we utilized data from quail ringings conducted in Italy (Toschi 1956, Sáez et al. 2023), specifically focusing on trajectories labeled as direct trips, return trips, reproduction 1 (pertaining to individuals ringed between March and May), and reproduction 2 (pertaining to individuals ringed between June and July). A collection of trajectories and nodes identifies a quail migratory network, and the route choices made to connect the different route segments. By comparing recoveries of ringed birds across time, we examined how changes in quail movements relate to changes: (1) in cities (indicated by the number of people, the number of graduates, and GDP); (2) in agrosystems (indicated by the areas under cereal and legume cultivation, and cereal and legume production volumes (ISTAT)); (3) in weather (indicated by the North Atlantic Oscillation (NAO)); and (4) in movement ecology (indicated by movement type and direction) (Nadal et al. 2019). In order to describe and compare migratory trajectories through time, we analyzed the average vector of quail movements (Nadal et al. 2022). We identified mega-urbanized areas using maps and assessed the number of trajectories crossing these areas. We predicted that, as trajectories cross mega-urbanized areas, their time-frequency would decrease and their directional movement would change (Fig. 1).

The quail’s annual cycle comprises four biological stages: wintering, spring migration (arrival), breeding period, and autumn migration (departure). Arrivals take place in February–April; the breeding period occurs in May–July; and departures take place in August–October (Alerstam and Bäckman, 2018; Sáez et al., 2023). The population demography of quail is linked to habitat quality by their movement patterns; birds initially settle in green areas to breed but abandon these for other breeding sites when the fields are harvested (Somveille et al. 2020; Willemoes et al. 2014). During the European summer, quail alternate between breeding activities and movement, searching for good-quality habitat and conspecifics. At the beginning of spring, quail breed in Africa and Mediterranean Europe, with subsequent breeding attempts in the Mediterranean and central Europe. Quail autumn migration begins in the middle of August, when they return to their African wintering grounds (Hadjikyriakou et al. 2020).

### Data collection

We employed a total of 4211 instances of recovery data spanning from 1939 to 1951, as documented by Toschi (1956). Additionally, we integrated 804 recovery records from 1998 to 2017 along with quail ringing data from the Italian Bird Ringing Scheme database located at ISPRA (Istituto Superiore per la Protezione e la Ricerca Ambientale). To ensure consistency, we processed and standardized these datasets, resulting in uniform information that encompassed details such as the specific day of the year, longitude, and latitude (Clark et al. 2009; Speek et al. 2010). We used Eurostat (https://www.eea.europa.eu/data-and-maps/dashboards/land-cover-and-change-statistics) to obtain the percentage occurrence of urbanized areas in each locality. Given that this study does not attempt to use ringing data to evaluate quail abundance and the fact that we studied routes that cross the Italian Peninsula, any differences in ring recovery effort should not affect our results. We used state time series data from ISTAT (Istituto Nazionale di Statistica, https://ebiblio.istat.it) and NAO (North Atlantic Oscillation, National Centers for Environmental Information, https://www.ncei.noaa.gov/access/monitoring/nao/).

According to the biological gap between the quail’s ringing and recovery (Spina and Volponi 2008), which was primarily caused by hunting, we divided trajectories into eight groups: (a) stopover (less than 5 days between ringing and recovery); (b) sedentary (ringing and recovery at the same site and not studied); (c) wintering (recovery between September and February); (d) direct trip (less than 180 days between ringing and recovery); (e) return trip (more than 180 days between ringing and recovery). Following Nadal et al. (2022), reproduction was divided into three sub-categories: reproduction 1 (ringing between March and May); reproduction 2 (ringing between June and July); and reproduction 3 (ringing between August and September). Four groups (direct trip, return, reproduction 1, and reproduction 2) by two directions (North and South) accounted for this study (Table 1). The recovery rate is calculated by dividing the count of previously ringed quails by the total number of quails initially ringed within a particular year. It is worth noting that a significant portion of these recoveries are reported by hunters.

**Table 1:**
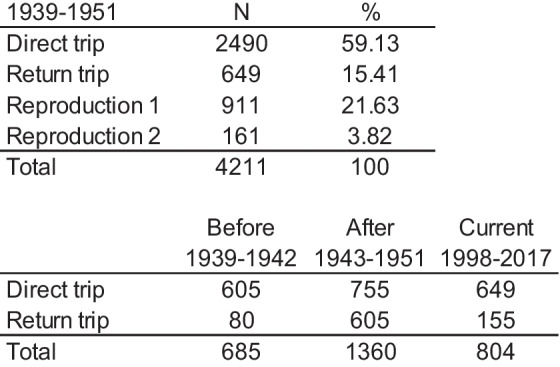
Quail recoveries between 1939 and 1951 (Toschi, 1956) and from the Italian Bird Ringing Scheme between 1998 and 2017

*Reproduction 1*, birds ringed March–May; *Reproduction 2*, birds ringed June–July

### Statistical analysis

We plotted the routes connecting the points where the quails were ringed and where they were subsequently found. These routes were categorized as either northerly (between 0 and 90° as well as 270° and 360° azimuth) or southerly (between 90 and 270° azimuth). We determined the average direction for each category by calculating the directional vector based on all included routes (Sáez et al. 2023). Within each category, we utilized the Rayleigh test to assess the uniformity of the taken directions. The test aimed to determine if there was a prevailing direction or if the routes were uniformly distributed (null hypothesis). We then compared these directions against a unidirectional trend (a single preferred direction). Additionally, we employed Hotelling’s paired test to evaluate differences between trajectories in each category. This test aimed to ascertain whether there were significant differences between the two directions. The null hypothesis here was that no significant difference existed between the directions. For conducting these statistical analyses, we used Oriana (Kovach Computing Services 2011).

We calculated generalized linear models (GLMs) with normal distributions and identity links, in which the number of quail recoveries varied according to the links between socioeconomic, agricultural, and climatic parameters and movement ecology (period of recovery, months), cities (GDP, the numbers of people, graduates, and hunters), agrosystems (area under wheat 〈km^2^〉, area under legumes 〈km^2^〉, area under cereals and legumes 〈km^2^〉, wheat production, tonnes, legume production, tonnes, cereal and legume production, tonnes (ISTAT)), and weather (monthly NAO, annual average NAO, and November–April NAO, measured in atmospheres). We applied a backwards stepwise model selection process in which the most parsimonious model was derived by systematically removing potential explanatory variables from the full model described above (where additional factors no longer contributed to statistical significance), and then calculated the parameter estimates and their standard deviations. The number of factors and the dataset (*N*≥25) were always balanced. The AICc (corrected Akaike information criterion), ΔAICc, and Akaike weights were used to select models (Burnham and Anderson 2002). We compared the relative merits (contributions of predictors) of each model to the other models. We used GLMs with a binomial function and a logit link to evaluate: (1) the differences between before the WWII period (1939–1942) and after the WWII period (1943–1951) regarding direction (north and south) and movement (direct trip, return trip, and reproductions 1 and 2); and (2) the differences between the periods (before and after WWII, and current) regarding direction (barrier, north, and south) and movement (direct trip and return trip).

Statistical analyses were performed using JMP16 (SAS Institute Inc 2021), Arcgis 10.8.1, and Oriana 4.02. We drew pre- and post-WWII trajectories for the routes taken on direct trips, return trips, reproduction 1, and reproduction 2. We synthesized the data on the north and south trajectories, and trips which crossed barriers to produce frequencies, standard deviations, and representations of their distributions. We prepared summary diagrams outlining route directions and angles with respect to north and compared the current period directions with those before and after WWII.

### Conceptual land models

**Fig. 2:**
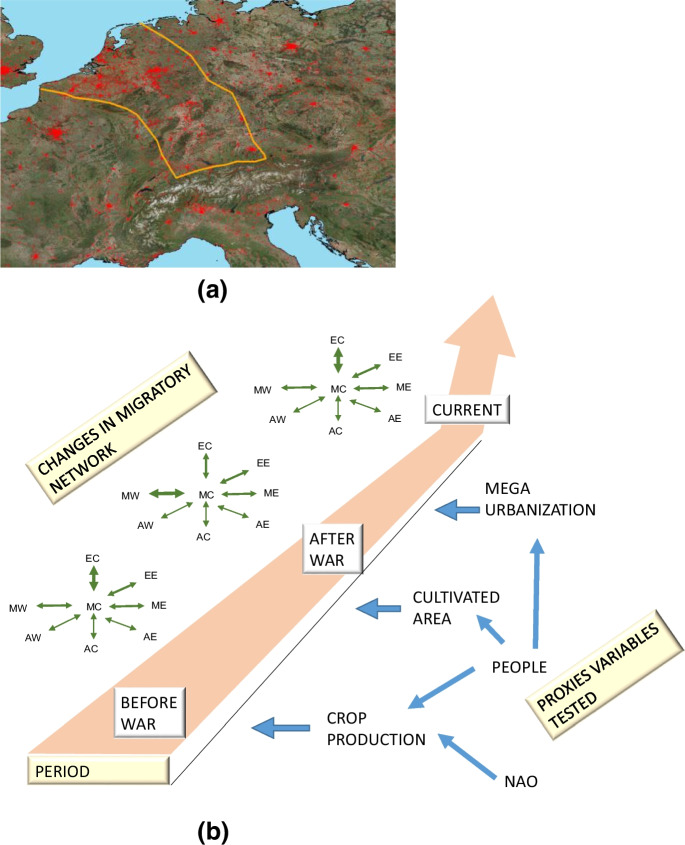
(**A**) Conceptual map representing European barrier: urbanized in red; perimeter of the mega-urbanization area in orange. (**B**) Conceptual model depicts the variables that affect the migratory network of quail as well as changes in the network through time. MW, Mediterranean West; AW, Africa West; EC, Europe Central; MC, Mediterranean Central; AC, Africa Central; EE, Europe East; ME, Mediterranean East; AE, Africa East

Migratory populations face various challenges throughout their journeys. A conceptual model can unravel the intricate relationship between quail and ecosystems during migration. This model elucidates the multifaceted factors influencing quail migration networks. Quail migrate to central Europe for their reproductive cycle. The Scheldt, Rhine, and Meuse lowlands in Europe provide optimal settings for quail reproduction, but these areas are now heavily urbanized. The emergence of a 300-km-wide barrier of mega-urbanization stretching from the North Sea to the Alps poses significant obstacles to their migration pathways, impeding their movement and altering their traditional routes (Fig. 2).

**Fig. 3:**
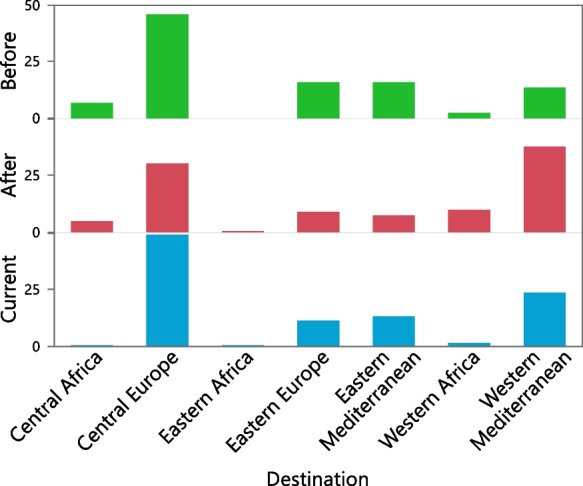
Quail dataset; percentage of recoveries of ringed quail with destinations outside Italy for the periods before and after WWII, and the current period

According to our data, we hypothesized that the quail migration pattern changed between the pre-WWII era and the post-WWII and contemporary eras. The central European node accounted for 34.9% of the endpoints of quail trips from Italy to foreign destinations (Figures 2 and 3), followed by the western Mediterranean (29.6%), African (13.03%), eastern European (11.44%), and eastern Mediterranean (10.37%) nodes. The rapid growth of mega-urbanization across central Europe has erected a significant artificial barrier, disturbing the migratory nocturnal patterns of quails. This urban impediment increases the likelihood risk of collisions between quail and infrastructures, posing threats such as the transmission of diseases through vector insects (Nadal et al. 2022).

Urbanizations have steadily evolved into mega-urbanizations since pre-WWII, as the human population has grown (Alberti and Wang 2022). Urban and global ecology, as interconnected disciplines, explore the intricate relationships among ecosystems, human societies, and wildlife. This comprehensive approach involves various levels and proxies to gauge the robustness of these associations. To illustrate the impacts of massive urban expansions and population surges on quail migratory networks, we have selected multiple indicators for city growth (such as GDP, population size, educational attainment, and number of hunters), agro-systems (including cultivated land and crop production), and weather patterns (such as the North Atlantic Oscillation—NAO) (Supplementary 3). By applying historical data to our model, we aim to discern the specific proxies that exert influence on migration pathways over time (Faaborg et al. 2010a).

## Results

### Trajectory direction and destination

**Fig. 4:**
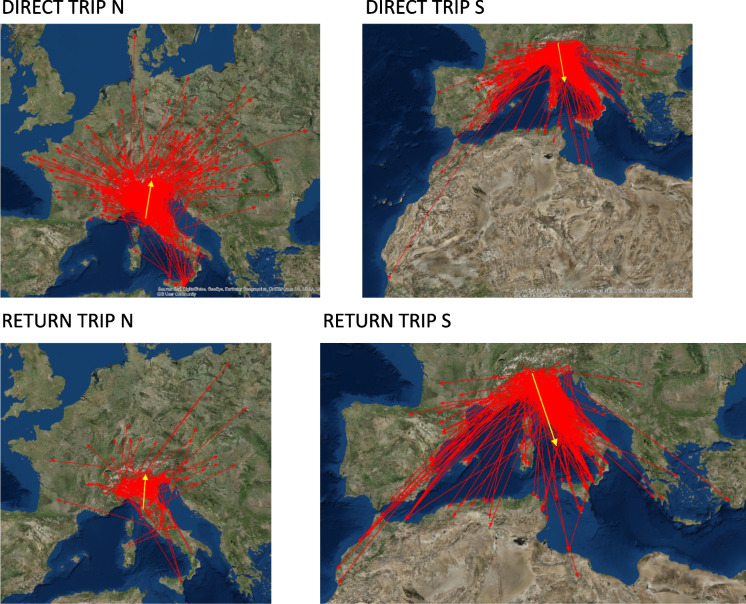
The migratory pathways of quail in each category. Red arrows show the trajectories of individual birds. Yellow arrows indicate the average trajectory. Direct trip N (north), direct trip S (south), return N (north), return S (south)

**Fig. 5:**
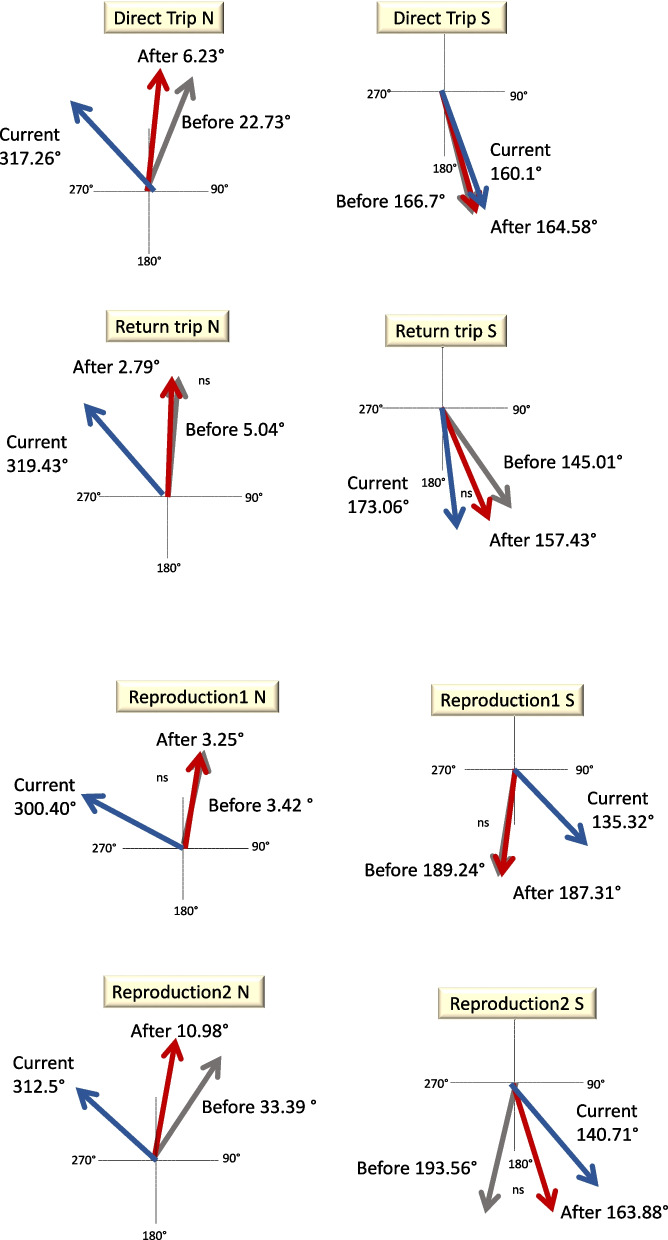
Representation of the average vector of quailmigratory trajectories (degrees of north) of groups across time. Before WWII (grey), after WWII (red), and current (blue). Direct trip N (north), direct trip S (south), return N (north), return S (south), reproduction 1 N (north), reproduction 1 S (south), reproduction 2 N (north), reproduction 2 S (south); ns, no significant Hotelling’s paired tests between the two arrows. Reproduction 1, birds ringed March–May; Reproduction 2, birds ringed June–July

Before WWII, direct trips (Figs. 4 and 5) in a northerly direction comprised 30.8% of the trajectories (Rayleigh test *Z* = 189.3, *P* < 0.0001, *N* = 605, with a mean direction = 22.3°±61.8° with respect to north), whereas trips in a southerly direction comprised 24.1% of the trajectories (Rayleigh test *Z* = 116.1, *P* < 0.0001, *N* = 474, with a mean direction = 166.7°±68° with respect to north). Return trips in a northerly direction comprised 4.4% of the trajectories (Rayleigh test, *Z* = 20.8, *P* < 0.0001, *N* = 86, with a mean direction = 5.04°±66.6° with respect to north), whereas trips in a southerly direction comprised 5.5% of the trajectories (Rayleigh test *Z* = 53.3, *P* < 0.0001, *N* = 107, with a mean direction = 145.1°±46.8° with respect to north).

Reproduction 1 individuals travelling in a northerly direction comprised 17.1% of the trajectories (Rayleigh test *Z* = 104.5, *P* < 0.0001, *N* = 336, with a mean direction = 3.4°±62° with respect to north), and those with a southerly direction comprised 13.5% (Rayleigh test *Z* = 71.1, *P* < 0.0001, *N* = 266, with a mean direction = 189.2°±65.8° with respect to north).

Reproduction 2 individuals travelling in a northerly direction comprised 2.2% of the trajectories (Rayleigh test *Z* = 13.4, *P* < 0.0001, *N* = 43, with a mean direction = 33.4°±61.8° with respect to north), and those in a southerly direction comprised 2.4% of all trajectories (Rayleigh test *Z* = 13.5, *P* < 0.0001, *N* = 48, with a mean direction = 193.6°±64.5° with respect to north).

### Pathways before and after the Second World War and in the present

Statistically significant differences were found within categories between the before and after WWII northerly trajectories (Figures 4 and 5); direct trips (Hotelling’s paired test, *F* = 4.4, *P* < 0.013, *N* = 1310); and reproduction 2 trips (Hotelling’s paired test, *F* = 3.9, *P* < 0.03, *N* = 80). Similar results were found within return trips for the before and after WWII southerly trajectories (Hotelling’s paired test, *F* = 4.4, *P* < 0.013, *N* = 1310) and reproduction 2 trips (Hotelling’s paired test, *F* = 3.9, *P* < 0.03, *N* = 80). The differences between the directions taken before and after WWII and the contemporary trips showed significant differences, except for the comparison between southerly return trips after WWII and currently (Fig. 5, Supplementary 1).

**Fig. 6:**
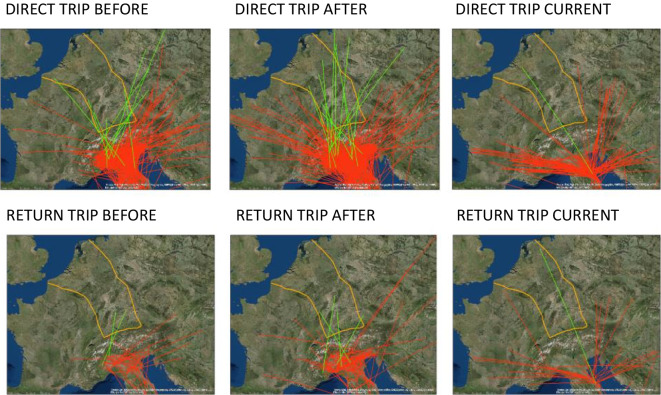
Quail migratory trajectories on direct and return trips, considering the periods before- and after-WWII, and current. Green: trajectories across the mega-urbanization barrier. Red: other trajectories. Orange: the perimeter of the mega-urbanization barrier

The recovery rate increased over time. The best GLM model (normal, identity) for explaining recovery rate included the following explanatory parameters, ordered by their importance as significant effects: period (before and after WWII, current); cereal and legume production; and the number of people (χ^2^ = 21.3, *P* < 0.0003, *N* = 40, AICc = −293, Supplementary 2). The GLM (binomial, logit) for the effects of periods (before and after WWII, current) were significantly explained by direction: north, south, and barriers (Fig. 6), and movement type: direct trip, return trip (χ^2^ = 933, *P* < 0.0001, *N* = 2849, AICc = 3010, deviance *P* < 0.0001). The binomial logit GLM model was employed to analyze periods before and after WWII. The model’s indicated factors included direction (north and south) and movement type (such as direct trip, return trip, reproduction 1, and reproduction 2), all of which were identified as significant effects (χ^2^ = 251, *P* < 0.0001, *N* = 4211, AICc = 5568, deviance *P* < 0.0001).

## Discussion

### Changes in migration routes

The study of the spatial and temporal consequences of historical changes on wildlife populations provides an effective method to evaluate the effects of human activities on natural patterns (Alberti et al. 2018). As our paper makes clear, the effects of the WWII destruction of towns and natural areas were felt by ecosystems (Gaynor et al. 2016). As demonstrated by our data, WWII had a minimal impact on the quail migratory network and did not affect every type of movement (Figs. 3 and 4). This is because migratory bird behavior has evolved to exploit a variety of diverse routes (Chen et al. 2021; Huang et al. 2022; Xu et al. 2022). A variety of migration routes can create a network of alternative migratory paths, enabling birds to successfully deal with unforeseen environmental or weather issues and reduce the resulting disturbance on population processes (Chernetsov and Markovets 2022; Ćiković et al. 2021).

The shortest route is not always the best option; detours and low-cost routes can be helpful if birds have a tailwind or can overcome barriers (Kranstauber et al. 2015). Migratory behavior varies greatly between individuals (Kürten et al. 2022). Other Afro-Palearctic migratory birds (Deboelpaep et al. 2022) have a similar migratory network to quail. A migratory network could help to explain the migratory divide of barn swallows (*Hirundo rustica*) in America (Turbek et al. 2022) and China (Hobson et al. 2015) as well as Swainson’s thrushes (*Catharus ustulatus*) in America (Justen et al. 2021). This plasticity in the chosen route is a response to environmental conditions (Verhoeven et al. 2022) that could reveal different migration strategies in different subspecies (Chan et al. 2022). Stored energy reserves and tailwinds influence route selection (Purcell and Brodin 2007), but the route chosen may influence a species’ population dynamics (Cohen et al. 2017). As our findings suggest, natural barriers condition the routes (Feng et al. 2021) and habitat loss acts as a barrier (Jia et al. 2021; Tankersley 2004).

Our results indicate that the migratory network of quail has changed across time, shifting toward north and southward trajectories from west and eastward ones. These changes can be explained as a response to the various environmental changes post-WWII, such as global warming, mega-urbanization, habitat loss, and  expanding human population. Afro-Palearctic birds must face mountain, sea, and desert barriers. Quail use tailwinds and night flights to cross barriers (Lopez-Ricaurte et al. 2021; Vansteelant et al. 2021). Therefore, as a component at the secondary level ALAN from cities represents a significant barrier which must be overcome (Burt et al. 2023; van Doren et al. 2021) because the extraneous light influences a bird’s directional orientation (Wiltschko et al. 2010). In line with this, our findings showed statistically significant differences in network directions before- and after-WWII compared with the present period. In addition, the GLM models indicated the differences between periods in the direction of travel and in relation to urban barriers.

### Causes and consequences of changes in migration routes

The decline in functional connectivity, attributed to habitat loss driven by expanding urbanization, has led to the degradation of migratory networks. This deterioration potentially poses adverse effects on population size (Xu et al. 2019) and overall health (Nadal et al. 2022; Yin et al. 2022). Mega-urbanization is proceeding at pace, both in the global North and South (Lauermann 2018) and the increase in associated physical structures is causing new pressures on wildlife (Jacobson et al. 1955; Nghiem 2015). Anthropogenic disturbance impacts the airspace, the ephemeral habitat of migration, (Diehl 2013), and buildings, towers, power lines, wind-farms, aerial devices, and pollution affect and modify the aerial connectivity between bird habitats (Lambertucci et al. 2015; Schwemmer et al. 2022; Zuluaga et al. 2021). Clearly, mega-urbanization and global warming may explain the observed changes in migratory networks (Lauermann 2018). Altering the sense and direction of bird movements may also change ecosystem dynamics and decrease biodiversity.

The biodiversity crisis could be solved by implementing a “no net loss” policy (Kujala et al. 2022). Because cities are reshaping biodiversity (Alberti and Wang 2022) and causing permanent wildlife losses (Aronson et al. 2014; Williams et al. 2020), we must better understand how to make them more permeable. Mega-urbanization stands as a significant impediment to achieving sustainable habitat development (Elmqvist et al. 2018; McDonald 2008). Urban design strategies must prioritize mitigating their adverse effects on nature (Pickett and Zhou 2015) by addressing issues such as noise (de Camargo Barbosa et al. 2020; Huet des Aunay et al. 2014), pollution (Pickett et al. 2016), infrastructure expansion (Ramaswami 2020), land consumption (Hahs 2016; Seto and Pandey 2019), and other environmental discharges (Girardet 2020; Zimmerer et al. 2021).

The pervasive influence of artificial light at night (ALAN), noise pollution, and increasing temperatures due to urbanization profoundly disrupt habitat connectivity (Challéat et al. 2021; Horton et al. 2019; Wilson et al. 2021). Among the pivotal attributes affecting animal populations, distribution, abundance, and connectivity reign paramount (Taylor et al. 1999). However, the emergence of mega-urbanization as a novel artificial barrier complicates the nocturnal migratory patterns of birds, altering their distribution (Gaston et al. 2013; Sánchez de Miguel et al. 2022; van Doren et al. 2017). Our findings suggest that mega-urbanization profoundly reshapes migratory routes and network (McLaren et al. 2018).

The escalating disruptions caused by mega-urbanization represent a global concern (Xue et al. 2020). Infrastructure expansion, agricultural intensification, and the looming specter of global warming collectively contribute to habitat loss, degradation, fragmentation, and barrier effects (Cabrera-Cruz et al. 2018, 2020; Sierro and Erhardt 2019). These factors, in turn, intricately influence population dynamics, connectivity, and the overall distribution of wildlife (Korpach et al. 2022; La Sorte and Horton 2021).

Urgent action is needed to enhance city permeability, facilitating successful bird migration across urban landscapes. This improvement not only fosters the survival of various species but also mitigates the spread of diseases (Jiménez-Peñuela et al. 2021). Our findings show that increasing human populations, consumption, global warming, and artificial barriers alter migratory patterns (Faaborg et al. 2010a, b) and increase the possibility of disease transmission (Alberti and Wang 2022; de Angeli Dutra et al. 2021; Kheirallah et al. 2021; Shestopalov et al. 2022). Connectivity plays a crucial role in population genetics and dynamics (Liu et al. 2022; Mudrik et al. 2022). Man-made structures such as buildings, towers, cranes, power lines, fences, wind turbines, and artificial light at night (ALAN) pose a significant threat to avian life, causing the loss of millions of birds annually (Burt et al. 2023; Loss et al. 2015). The historical journey of quail migration illustrates the anthropogenic impact on ecosystems and biodiversity, revealing intricate links between various biomes. Improved urban planning can reduce the negative consequences of mega-urbanisations. Changes in migratory patterns underscore the importance of adopting a One Health for One Planet paradigm. This necessitates a multidisciplinary approach that harmonizes human well-being with the conservation of biodiversity

## Conclusions

Throughout history, ecosystems and animal populations have undergone significant changes. Before and after the Second World War, as compared to the present day, the migration pattern of quail has shifted due to the increase in urbanized land and environmental evolution. Current artificial ecosystems act as barriers that affect animal migrations and impact their populations. Economic activity metrics, such as cereal and legume production and population numbers, are intertwined with the growth of cities as well as the characteristics of wildlife migration patterns. The routes of quail migration are disrupted by the increase in economic activity and city size. Quail, like other migratory birds, may adapt their migration routes and timings in response to these changes. Such adaptations are a survival strategy to find suitable habitats and resources.

The well-being and health of people depend on how we utilize and transform the land. As urban belts expand, the likelihood of disease transmission from wildlife to society increases, simultaneously leading to the depletion of wildlife resources and ecosystem services. Consequently, there arises an urgent  need for a comprehensive approach to urban development that considers its effects on the environment, wildlife, and human welfare. This study recognizes the interdependence of urban development, the natural world, and the societal well-being, promoting a harmonious coexistence between urban areas and nature, which is essential for ensuring the long-term health and quality of life for both humans and the planet as a whole.

### **Supplementary information**

ESM 1 (PDF 88.0 kb)

ESM 2 (PDF 48.0 kb)

ESM 3 (PDF 296 kb)

ESM 4 (PDF 660 kb)

ESM 5 (PDF 620 kb)

## Data Availability

We are unable to disclose specific data due to confidentiality agreements and data usage restrictions.

## Declarations

All authors have read, understood, and have complied as applicable with the statement on “Ethical responsibilities of Authors” as found in the Instructions for Authors.
