# Supplementary figures and images for: The effects of cities on quail (Coturnix coturnix) migration: a disturbing story of population connectivity, health, and ecography
Source: Environ Monit Assess. 2024 Feb 14;196(3):266. doi: 10.1007/s10661-023-12277-4 (PMC10867070; doi:10.1007/s10661-023-12277-4)

Supplementary 1

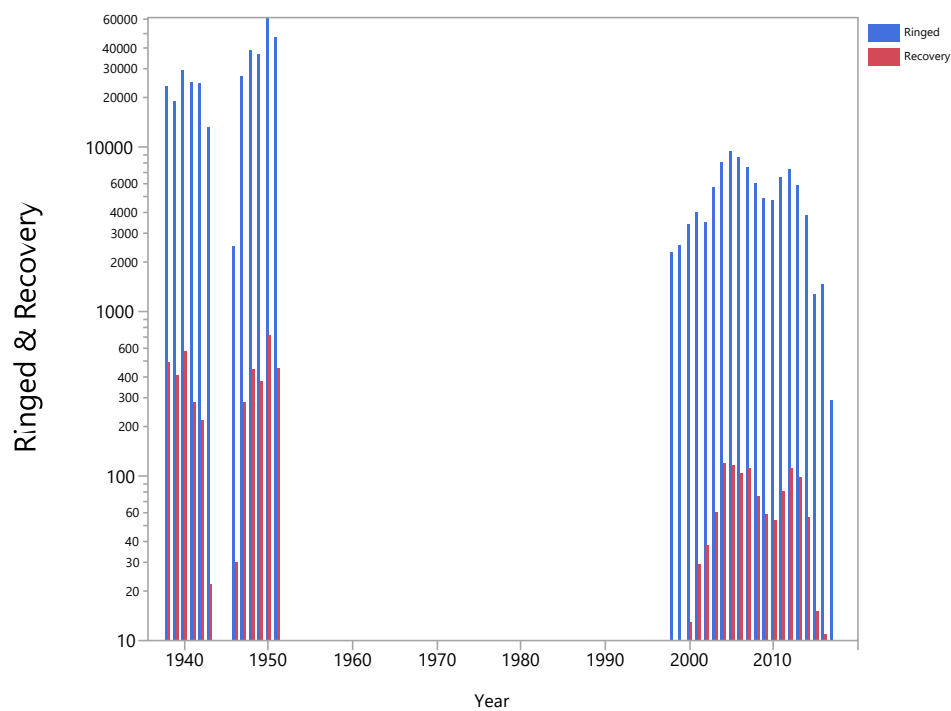

Supplement: Supplementary file 1 — (PDF 88.0 kb) [file 10661_2023_12277_MOESM1_ESM.pdf]

BEFORE DIRECT TRIP N

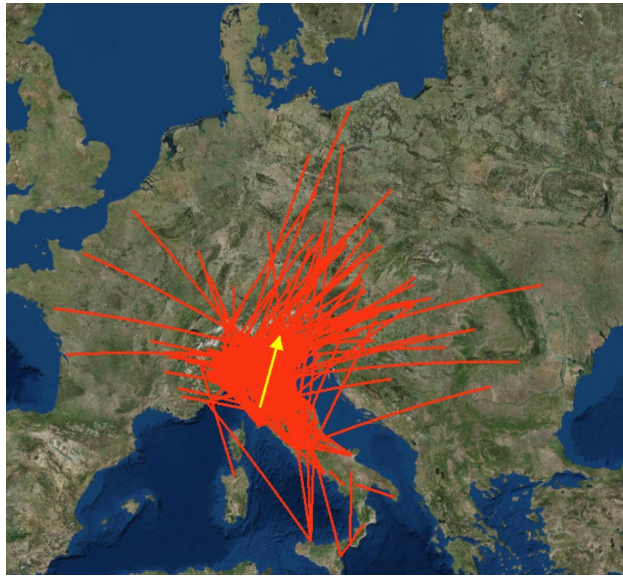

AFTER DIRECT TRIP N

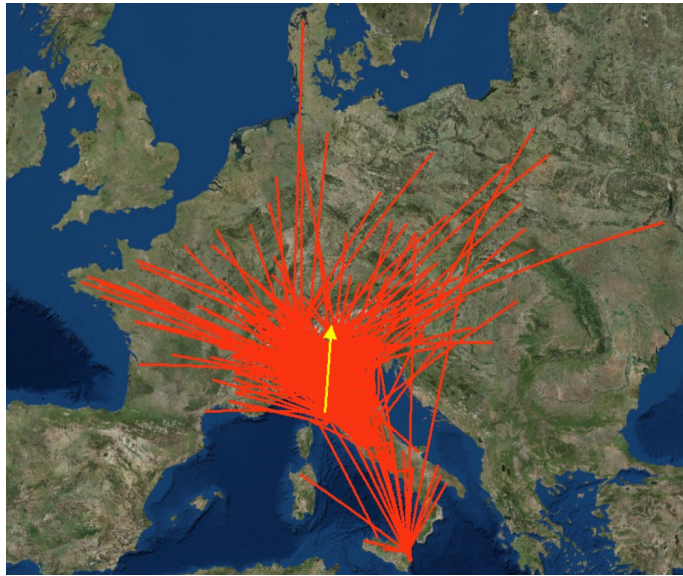

BEFORE DIRECT TRIP S

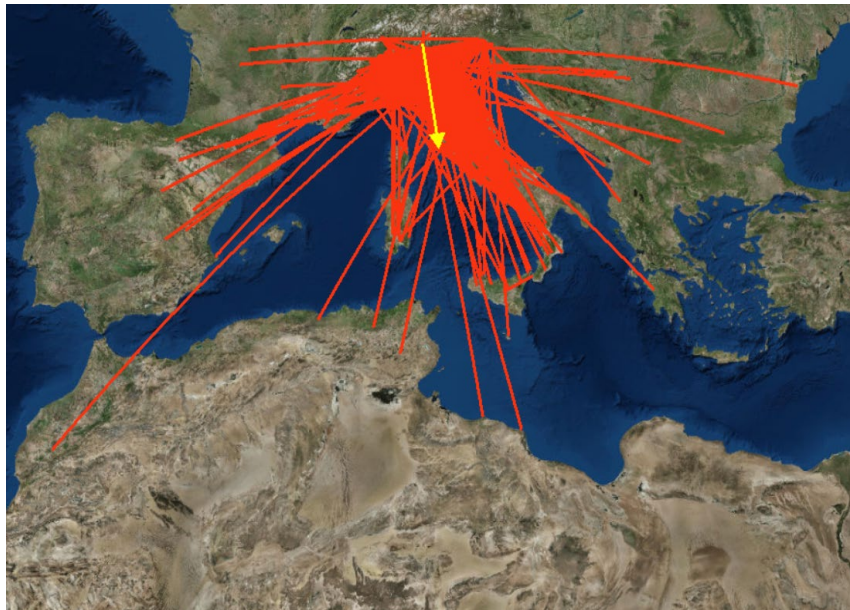

AFTER DIRECT TRIP S

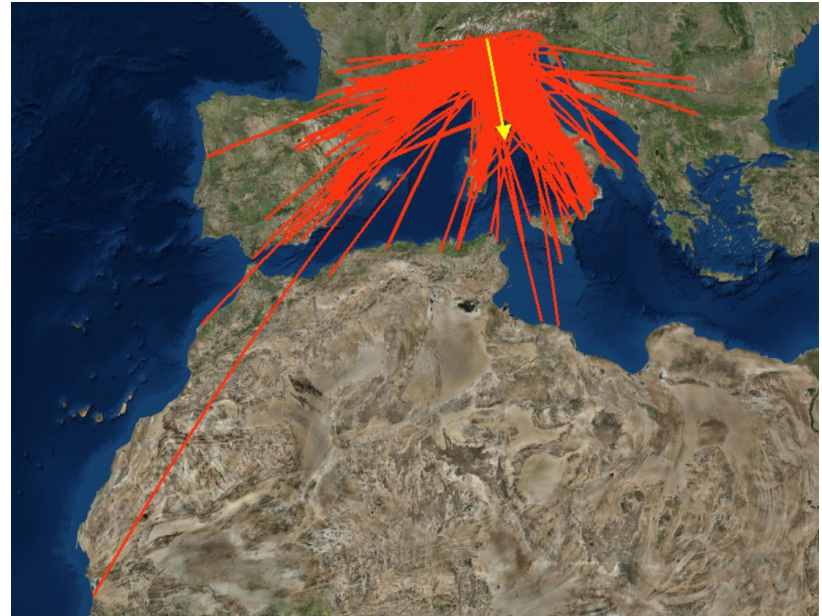

Supplement: Supplementary file 4 — (PDF 660 kb) [file 10661_2023_12277_MOESM4_ESM.pdf]
