# Supplementary material for: The effects of cities on quail (Coturnix coturnix) migration: a disturbing story of population connectivity, health, and ecography
Source: Environ Monit Assess. 2024 Feb 14;196(3):266. doi: 10.1007/s10661-023-12277-4 (PMC10867070; doi:10.1007/s10661-023-12277-4)
Supplement: Supplementary file 2 — (PDF 48.0 kb) [file 10661_2023_12277_MOESM2_ESM.pdf]

Supplementary 2

Rayleigh tests between the average vector of trajectories (degrees respecting North) in the periods before and after the war with the contemporary ones.

Constrats of trajectories before and after the war with the contemporary ones

Hotelling's paired test	F	P	N
Return N before-currently	7.07	0.002	157
Return N after-currently	5.48	0.006	209
Return S before-currently	6.97	0.002	191
Return S after-currently	0.1	0.905	402
Trip N before-currently	52.13	<0.0001	839
Trip N after-currently	51.9	<0.0001	989
Trip S before-currently	49.91	<0.0001	889
Trip S after-currently	70.82	<0.0001	1121
Reproduction1 N before-currently	54.29	<0.0001	427
Reproduction1 N after-currently	55.53	<0.0001	249
Reproduction1 S before-currently	17.79	<0.0001	321
Reproduction1 S after-currently	22.53	<0.0001	206
Reproduction2 N before-currently	13.85	<0.0001	81
Reproduction2 N after-currently	12.77	<0.0001	75
Reproduction2 S before-currently	9.36	<0.0001	92
Reproduction2 S after-currently	9.4	<0.0001	77
