# Supplementary material for: The effects of cities on quail (Coturnix coturnix) migration: a disturbing story of population connectivity, health, and ecography
Source: Environ Monit Assess. 2024 Feb 14;196(3):266. doi: 10.1007/s10661-023-12277-4 (PMC10867070; doi:10.1007/s10661-023-12277-4)

Supplementary 3

Best GLM model explaining recoveries includes the explanatory parameters ordered by their importance: period (before and after the war, current), cereal and legume production, and people.

Model	N	K	AICc	Delta AICc	AICc weight	Significant effects
1	40	3	-293	0	1	Period, cereal legume production, people
2	34	3	-242	51	8.42E-12	Cereal legume production, people
3	31	4	-236	57	4.19E-13	People, period
4	25	4	-181	112	4.78E-25	People, period
5	25	5	-178	115	1.06E-25	People, period

Model	Parameters included without significant effects
3	Production cereal legume, NAOnovember-april
4	Hunters, NAOnovember-april
5	Production cereal legume, Hunters, NAOnovember-april

Model: number of model (ordered by the most parsimonious)

N: number of data

K: number of parameters included in the model

AICc: corrected Akaike information criterion

Delta AICc: difference between corrected Akaike information criterion

AICc weight: Akaike information criterion weight

Significant effects: parameters included in the model with significant effects

Correlation between factors used in GLM

	Yeara	Naa	Recovery rate	GDP	Hunters	S weat	S cere legu	S herba	P wheat	P cere legu	P herba	People	Graduates
Yeara	1.0000	0.5045	-0.1190	0.0408	0.3768	0.0484	-0.0165	-0.1736	0.3389	-0.3546	-0.0879	0.0495	-0.2719
Naa	0.5045	1.0000	0.1288	0.2167	0.1436	-0.1637	-0.1946	-0.2782	0.3301	0.1014	0.1077	0.2722	0.0308
Recovery rate	-0.1190	0.1288	1.0000	0.2903	-0.3805	-0.1844	-0.1603	0.2436	0.2319	0.5285	0.2089	0.2440	0.4794
GDP	0.0408	0.2167	0.2903	1.0000	-0.1202	-0.9861	-0.9861	-0.6085	0.3574	0.3880	0.8536	0.9740	0.8551
Hunters	0.3768	0.1436	-0.3805	-0.1202	1.0000	0.0404	-0.1084	-0.4523	0.4833	-0.4680	-0.4421	0.1861	-0.4284
S weat	0.0484	-0.1637	-0.1844	-0.9861	0.0404	1.0000	0.9961	0.6697	-0.2849	-0.3122	-0.8592	-0.9774	-0.8574
S cere legu	-0.0165	-0.1946	-0.1603	-0.9861	-0.1084	0.9961	1.0000	0.6838	-0.3212	-0.2913	-0.8392	-0.9883	-0.8245
S herba	-0.1736	-0.2782	0.2436	-0.6085	-0.4523	0.6697	0.6838	1.0000	-0.0595	0.1301	-0.4888	-0.7131	-0.4842
P wheat	0.3389	0.3301	0.2319	0.3574	0.4833	-0.2849	-0.3212	-0.0595	1.0000	0.5251	0.2298	0.3781	0.1177
P cere legu	-0.3546	0.1014	0.5285	0.3880	-0.4680	-0.3122	-0.2913	0.1301	0.5251	1.0000	0.2766	0.3593	0.4838
P herba	-0.0879	0.1077	0.2089	0.8536	-0.4421	-0.8592	-0.8392	-0.4888	0.2298	0.2766	1.0000	0.7838	0.6987
People	0.0495	0.2722	0.2440	0.9740	0.1861	-0.9774	-0.9883	-0.7131	0.3781	0.3593	0.7838	1.0000	0.8298
Graduates	-0.2719	0.0308	0.4794	0.8551	-0.4284	-0.8574	-0.8245	-0.4842	0.1177	0.4838	0.6987	0.8298	1.0000

Yeara: annual NAO; Naa: November to April NAO; GDP: gross domestic product; Hunters: Number of hunters; S weat: surface cultivated with wheat; S cere legu: surface cultivated with cereal and legume; S herba: surfaced cultivated with herbaceous cultures; P wheat: production of wheat; P cere legu: production of cereal and legume; P herba: production of herbaceous cultures; People: number of humans; Graduates: individuals with a university grade

Dispersion graph of factors used in GLM

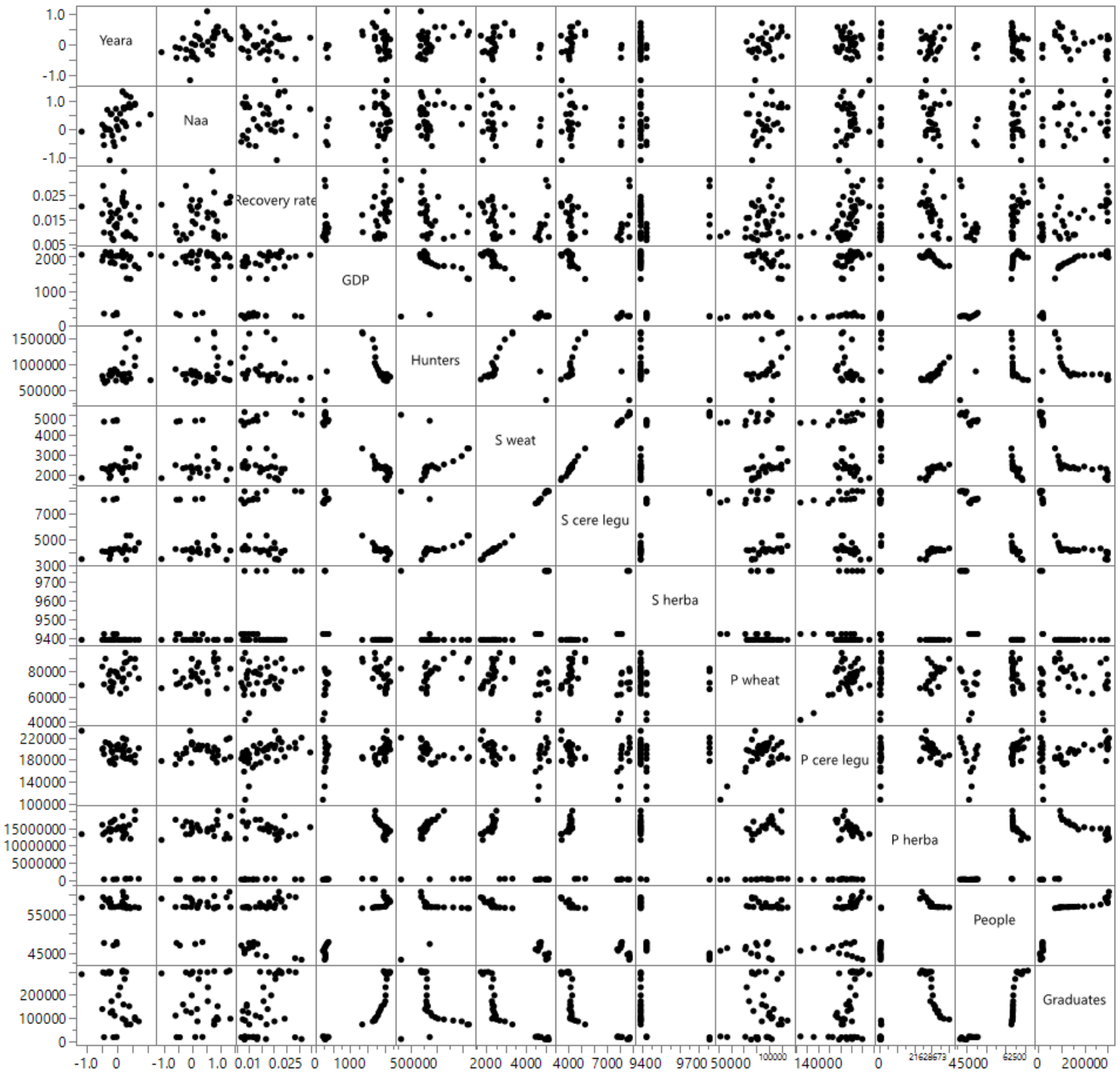

Supplement: Supplementary file 3 — (PDF 296 kb) [file 10661_2023_12277_MOESM3_ESM.pdf]
