# Supplementary material for: The effects of cities on quail (Coturnix coturnix) migration: a disturbing story of population connectivity, health, and ecography
Source: Environ Monit Assess. 2024 Feb 14;196(3):266. doi: 10.1007/s10661-023-12277-4 (PMC10867070; doi:10.1007/s10661-023-12277-4)

Supplementary 5

Historical photography Hengelo

Example of change in the urbanization of a city of the European plain.

Hengelo, 52.26° N, 6.78° E

current photo from Google Earth and photo from 1944-09-13 from Wageningen UR Library, Special Collections

https://library.wur.nl/WebQuery/geoportal/raf

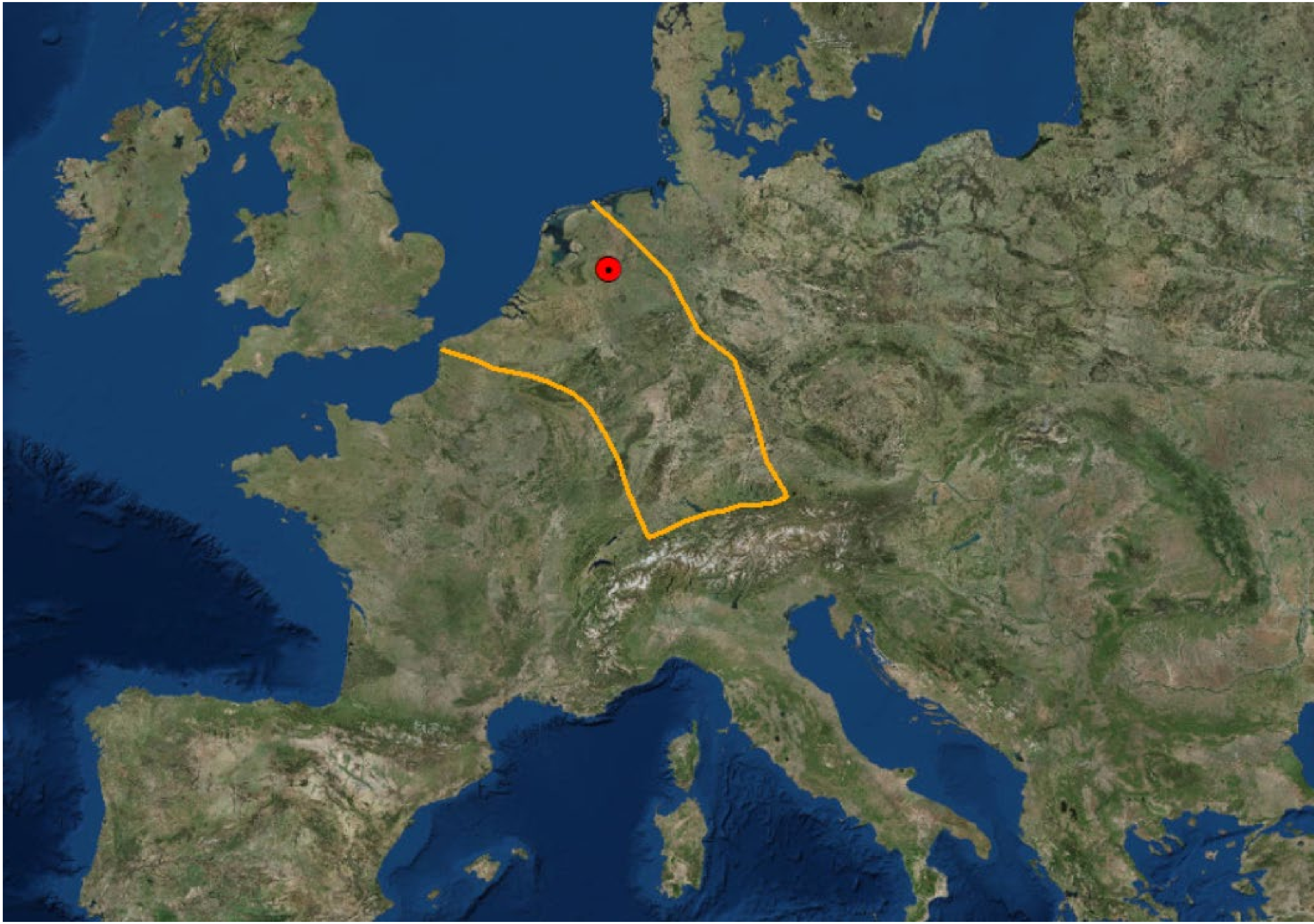

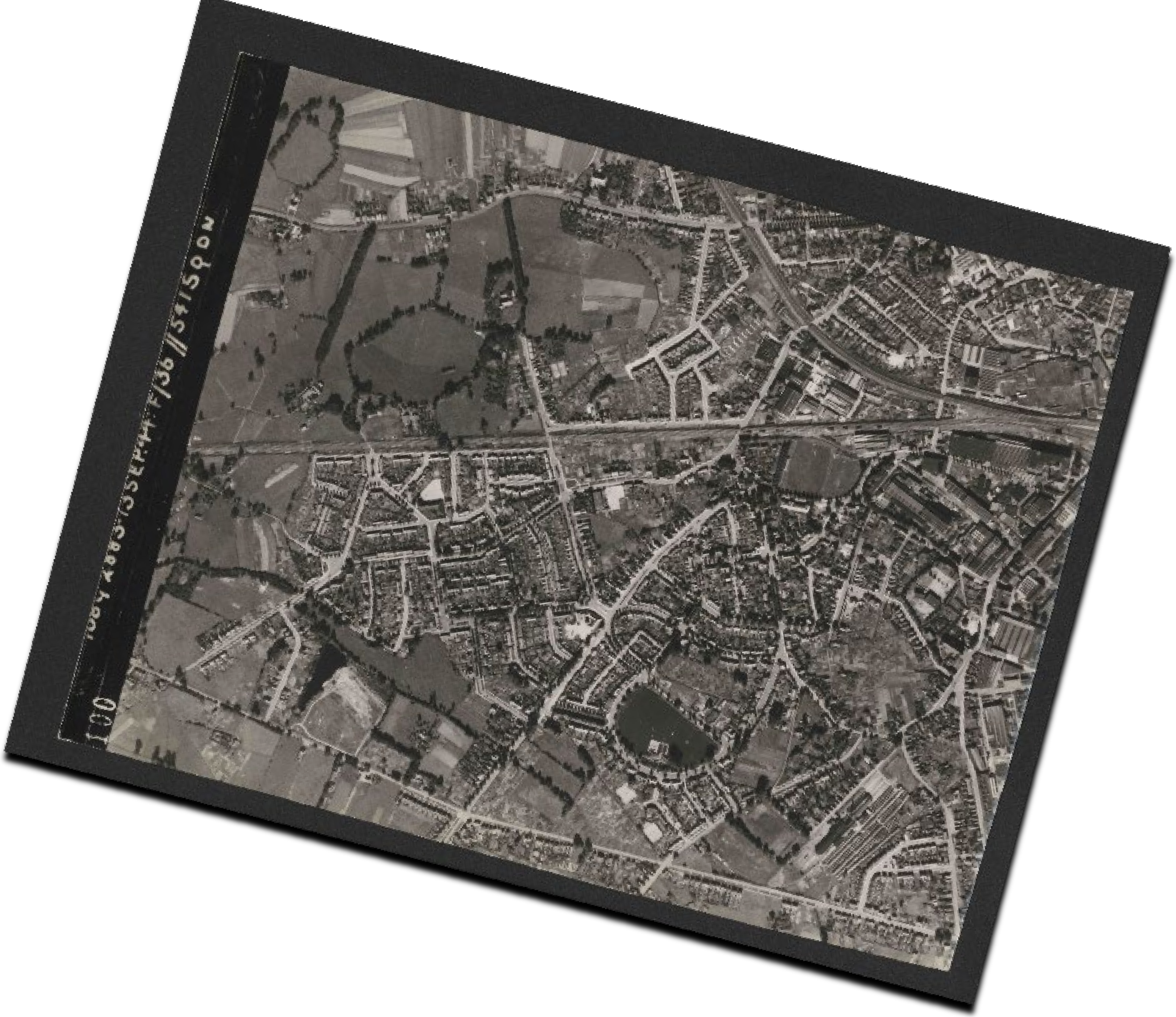
3SEP44 F/36 // 541SQDN
100

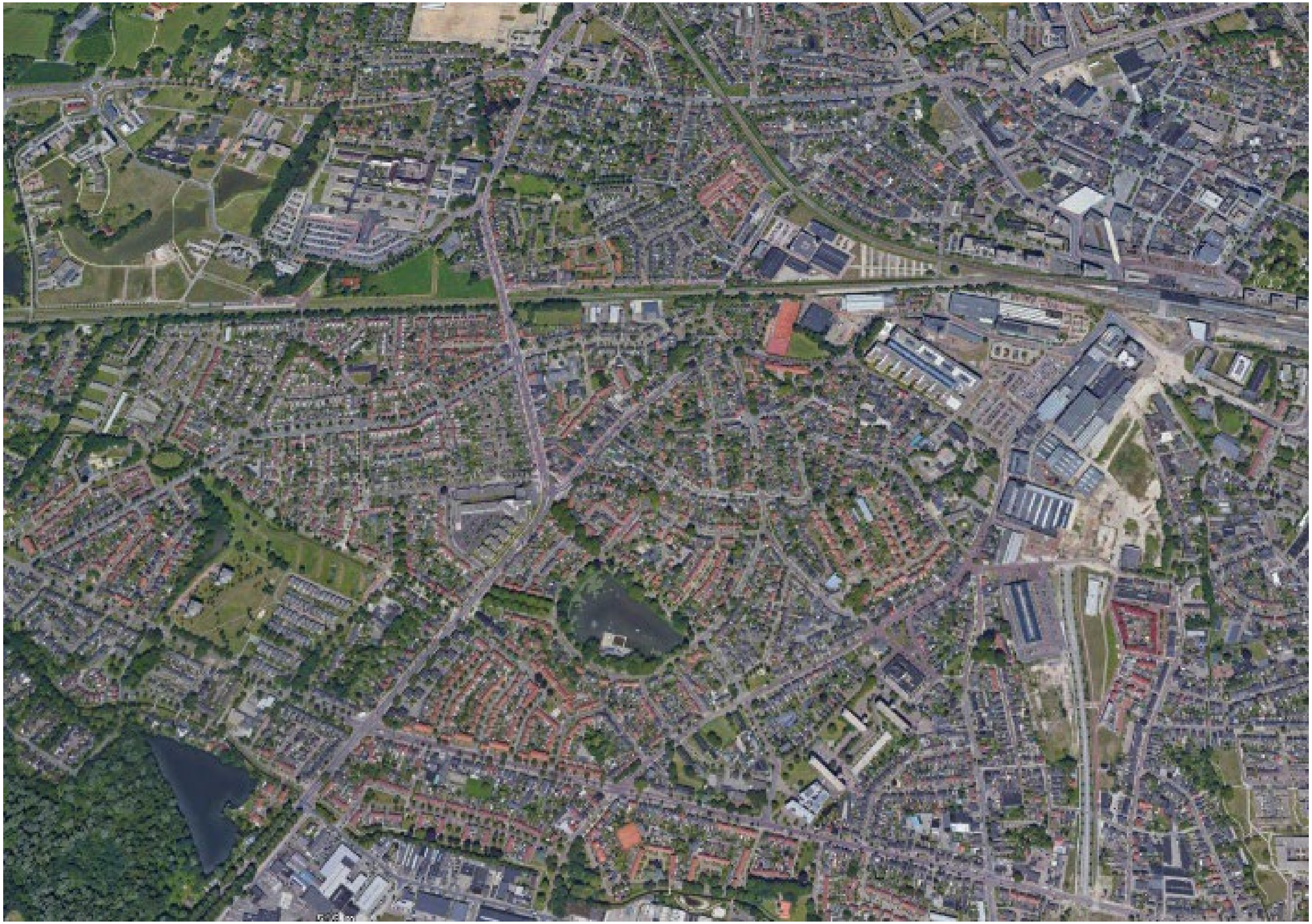

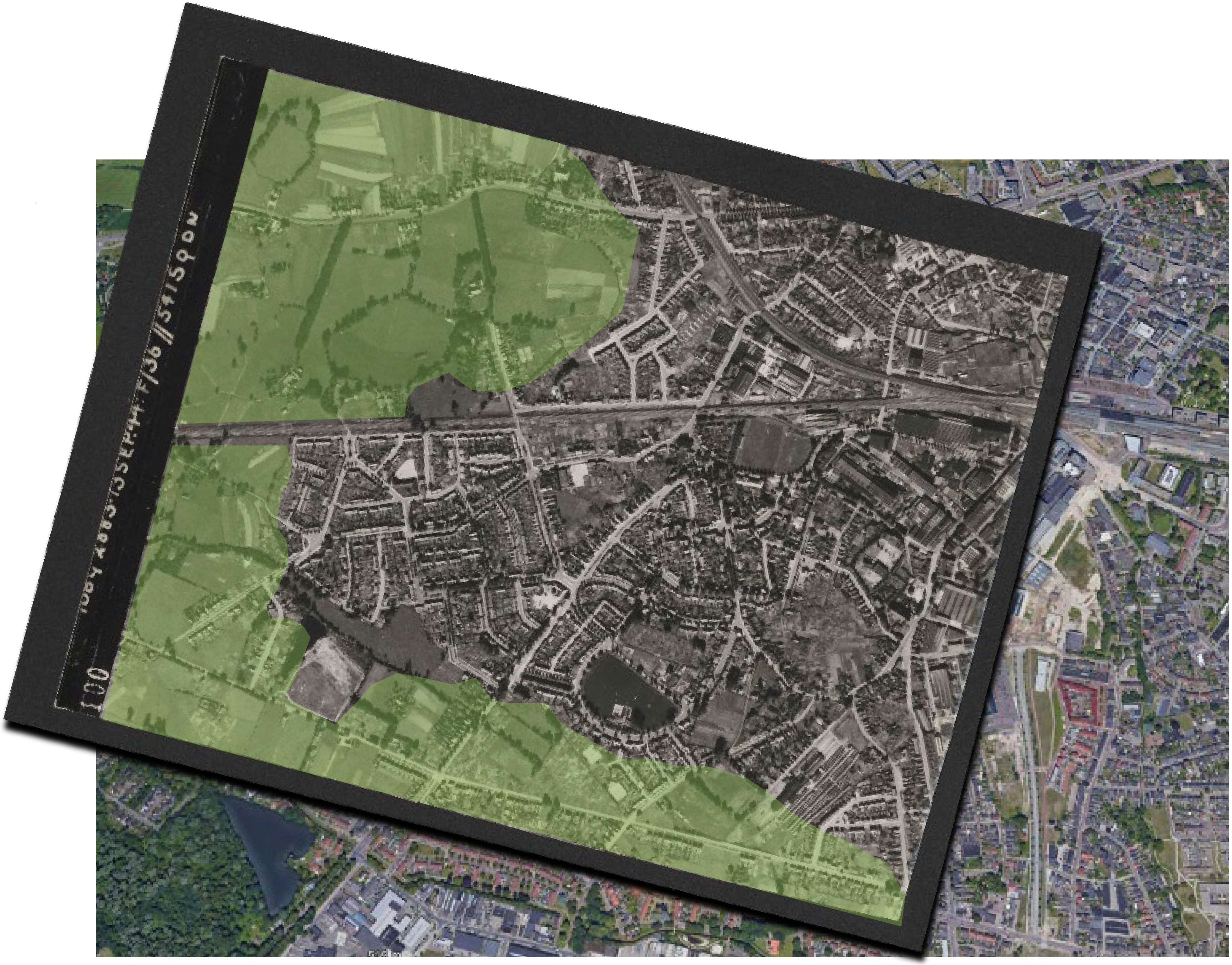

100
3SEP44 F/36 // 541 SQDN

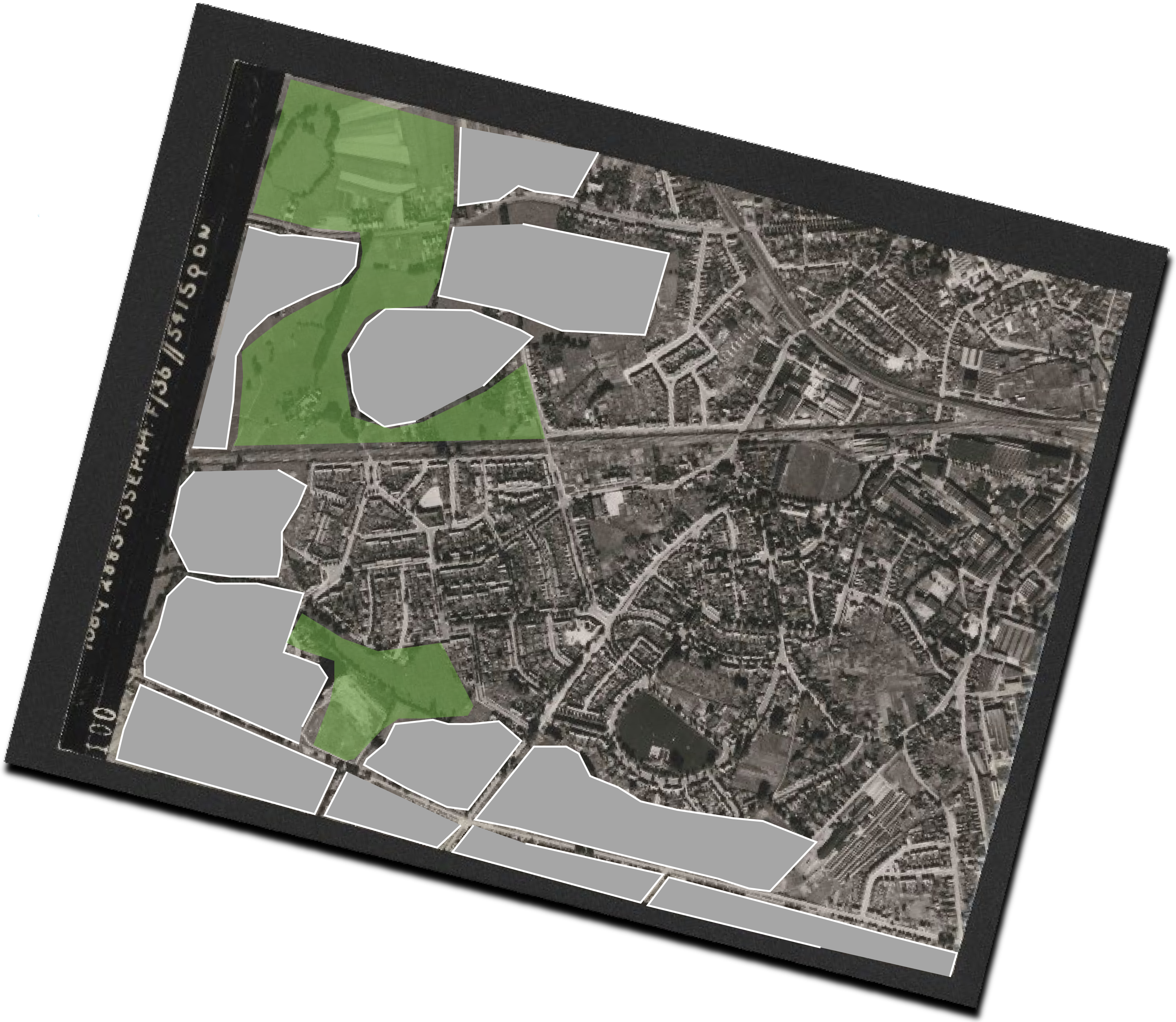

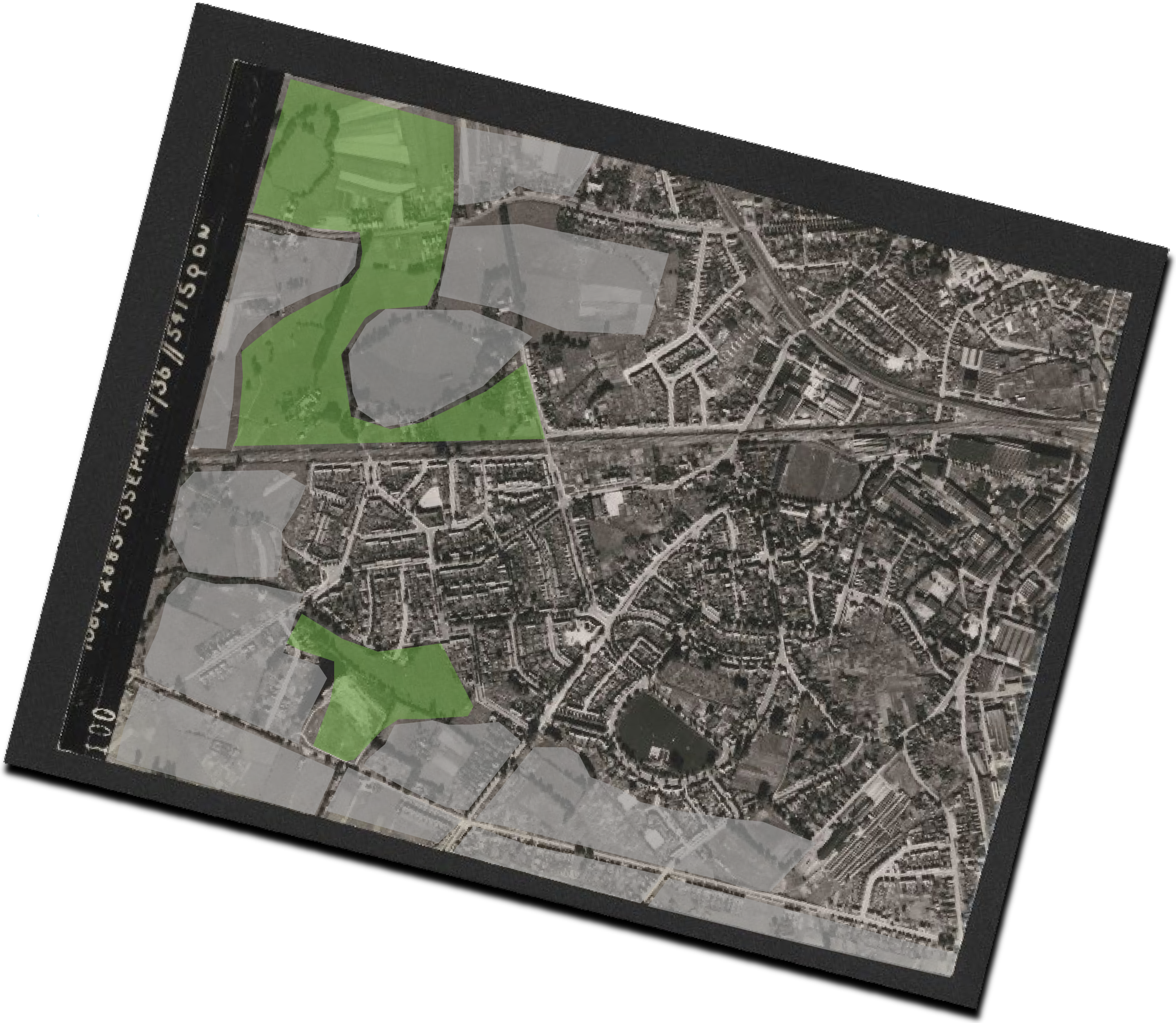
100

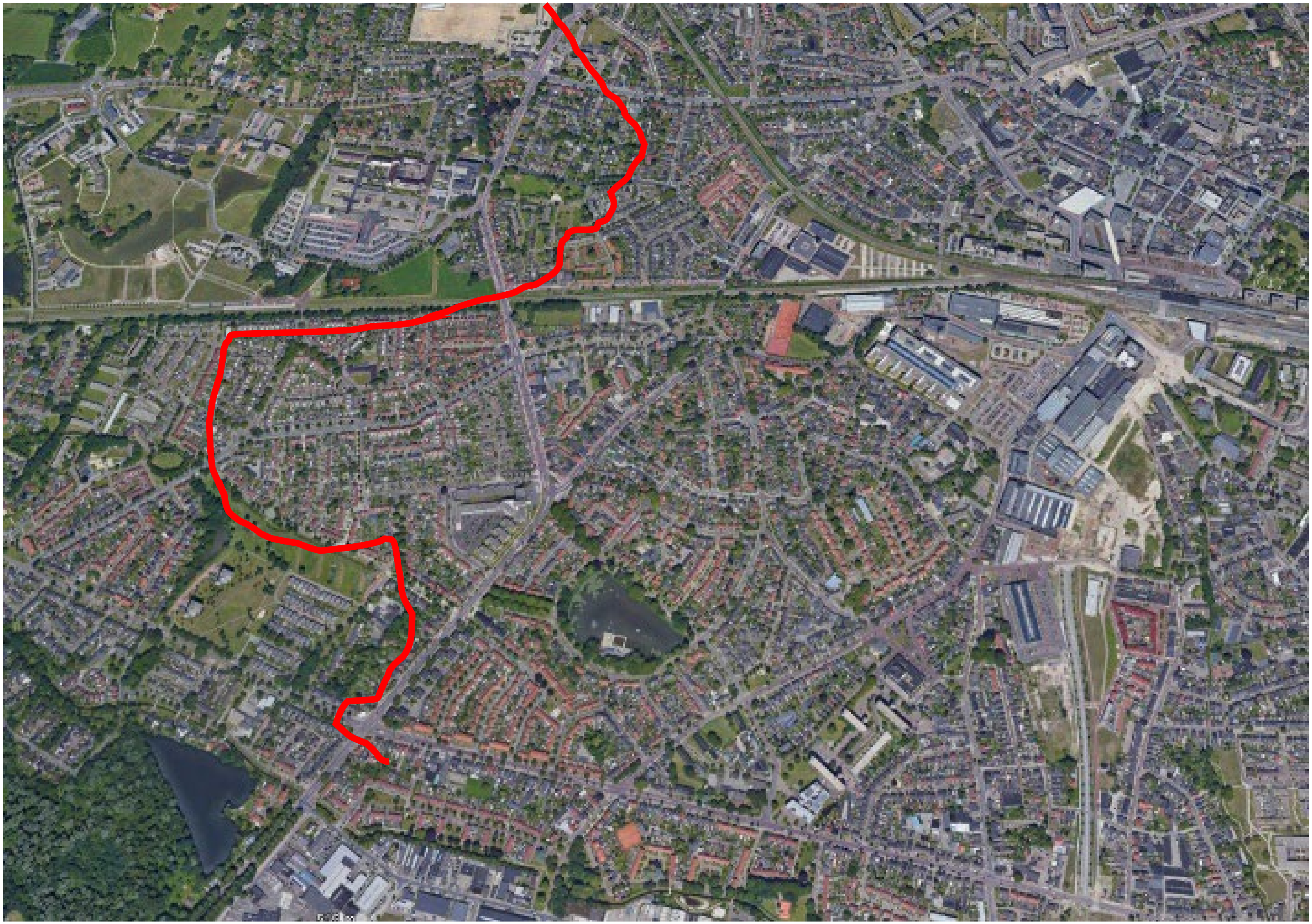

Supplement: Supplementary file 5 — (PDF 620 kb) [file 10661_2023_12277_MOESM5_ESM.pdf]
